# Effect of Cold Stress on Photosynthetic Traits, Carbohydrates, Morphology, and Anatomy in Nine Cultivars of *Stevia rebaudiana*

**DOI:** 10.3389/fpls.2018.01430

**Published:** 2018-09-28

**Authors:** Shokoofeh Hajihashemi, Fariba Noedoost, Jan M. C. Geuns, Ivica Djalovic, Kadambot H. M. Siddique

**Affiliations:** ^1^Plant Biology Department, Faculty of Science, Behbahan Khatam Alanbia University of Technology, Behbahan, Iran; ^2^Laboratory of Functional Biology, KU Leuven, Leuven, Belgium; ^3^Institute of Field and Vegetable Crops, Novi Sad, Serbia; ^4^The UWA Institute of Agriculture, The University of Western Australia, Perth, WA, Australia; ^5^UWA School of Agriculture and Environment, The University of Western Australia, Perth, WA, Australia

**Keywords:** carbohydrate, cold stress, morphology, photosynthesis, stem, *Stevia rebaudiana*, stomata

## Abstract

*Stevia rebaudiana* Bertoni is a sweet medicinal herb that is cultivated worldwide. This study aimed to identify the genotypic responses and function of nine cultivars of *S. rebaudiana* (accession numbers 1–9 from the EUSTAS Stevia Gene Bank) to low temperature. Plants were grown *in vitro* and incubated under controlled conditions at 5° or 25°C for 1 month. Cold stress significantly decreased the maximum quantum yield of photosystem II (F_v_/F_m_) in all cultivars, which was more pronounced in cultivars 5, 6, 8, and 9. The efficiency of photosystems I and II (PI_ABS_) also declined in cold-stressed plants and was accompanied by reductions in net photosynthesis (P_N_), intercellular CO_2_ (C_i_), water use efficiency (WUE), and chlorophyll *a*, chlorophyll *b* and carotenoid contents, more so in cultivars 5, 6, 8, and 9. Regardless of the downregulation of photosynthetic capacity, the cold stress increased water-soluble carbohydrates in all cultivars, which was accompanied by an increase in fresh leaf mass and area, more so in cultivars 5, 6, 8, and 9. Furthermore, cold stress increased the stomatal index and density, epidermal cell density, stem diameter, xylem vessel width, phloem tissue width, and number of sclerenchyma in all cultivars. Even though the nine cultivars of *S. rebaudiana* had lower PSII efficiencies at low temperatures, the increase in carbohydrates and leaf mass suggests that damage to PSII is not responsible for the reduction in its efficiency.

## Introduction

*Stevia rebaudina* Bertoni is a perennial sweet medicinal herb belonging to the Asteraceae family and native to Paraguay, Brazil, and Argentina with their subtropical climates and mild winters. *Stevia* leaves contain sweet diterpene glycosides or steviol glycosides (SVglys), which form up to 25% of the leaf dry mass (Geuns, 2003). Glycosyltransferase enzymes are responsible for glycosylation of the steviol molecule by transferring the glucose moiety to form various SVglys, with different units of D-glucose attached to the steviol ring. Large amounts of SVglys are found within the leaves of *S. rebaudiana* (Ceunen and Geuns, 2013). Accordingly, a noticeable amount of the carbon generated by photosynthesis is used for SVglys biosynthesis. *Stevia* cultivation is relatively successful in different regions of the world, though plants may experience cold stress in winter. Cold stress affects the physiological, biochemical, molecular, growth, and developmental processes of plants (Kumar Yadav, 2010). Plant exposure to low temperature during winter results in changes in photosynthetic capacity, plant anatomy, and carbohydrate contents (Ensminger et al., 2006; Cohu et al., 2014; Muller et al., 2014; Adams et al., 2016). Limited research has been undertaken on *Stevia* in relation to its performance under cold stress (Soufi et al., 2016) and no studies have sought to identify variation in *Stevia* cultivars in their acclimatory responses during growth in cold seasons.

Photosynthesis is the principal process of capturing light energy to form carbohydrates and is sensitive to low temperature (Adams and Demmig-Adams, 2004; Ensminger et al., 2006; Fan et al., 2015; Stewart et al., 2016). Chlorophyll (Chl) fluorescence is a direct tool for detecting photosystem II (PSII) efficiency, as the ratio of F_v_ to maximal fluorescence emission (F_v_/F_m_) (Logan et al., 2007; Adams et al., 2013, 2014). Although photoinhibition reduces efficiency of photosynthetic electron transport, it can be regarded as a means of protection of the photosynthetic apparatus in response to environmental stress; its key characteristics are a reduction in Fv/Fm and dissipation of light energy as heat. Despite the reduction in photosynthetic capacity it is often accompanied by increased sugar accumulation, which is a typical stress response in many plants (Adams et al., 2006, 2013, 2014; Logan et al., 2014). In *Vicia minor*, a reduction in photosynthetic rate and PSII efficiency in response to low temperature was accompanied by soluble sugar accumulation (Adams et al., 2013, 2014). Characterization of the PI_ABS_ is another tool that reflects the efficiency of both PSI and PSII in different conditions (Živčák et al., 2008). Indications of photosynthetic apparatus responses to environmental stress can also be based on variations in intercellular CO_2_ concentration (C_i_), P_N_, or WUE (Adams et al., 2013, 2014; Cohu et al., 2014).

Stomata play an important role in regulating photosynthesis and transpiration. Stomatal density is the number of stomata per unit leaf area, whereas the stomatal index is a function of both the number of stomata and the size of the epidermal cells. Stomatal density and index are ecophysiological parameters that can be affected by environmental factors such as drought, humidity, light, heavy metals, and CO_2_ concentration (Royer, 2001; Banon et al., 2004; Vezza et al., 2018). Numerous studies have shown that stomatal size, density, and pattern of stomatal distribution in leaves are controlled by long-term high temperatures (Maherali et al., 2002; Franks and Farquhar, 2007; Zheng et al., 2013; Hill et al., 2014; Yan et al., 2017; Wu et al., 2018). Photosynthetic capacity is closely linked to stomatal density (Xu et al., 2016). Wu et al. (2018) reported that warming had a different effect on two cultivars of *Syzygium*. High temperature significantly decreased stomatal size in *S. rehderianum* and stomatal density in *S. superba*. Warming also decreased photosynthesis in *S. superba*, but had no significant effect on photosynthesis in *S. rehderianum* (Wu et al., 2018). Wu et al. (2018) reported that a reduction in stomatal density had a negative effect on photosynthesis, while maintaining stomatal conductance and the photosynthetic rate was probably due to a reduction in stomatal size. Accordingly, investigating stomatal responses in the leaves of different *Stevia* cultivars experiencing low temperature may be important for gas exchange and photosynthetic capacity.

Understanding the changes in plant anatomy in response to cold stress will improve our knowledge of how plants cope with low temperature. Water transport is mainly related to the structure of the xylem vessels in plant stems (Banon et al., 2004). The geographical distribution of the plant may be relevant to the occurrence of narrow stem vessels, which provide high resistance to cavitation from low temperatures during winter. Vessel grouping in the stem protects against cavitation (Psaras and Sofroniou, 2004). Several studies have reported significant increases in the number of phloem cells in leaf veins in response to low temperature, which increased sugar export from leaves to other parts of the plant (Cohu et al., 2014; Muller et al., 2014; Stewart et al., 2016). Furthermore, cold stress can increase leaf thickness and dry mass in some plant species (Adams et al., 2013; Cohu et al., 2014; Stewart et al., 2016).

Different plant cultivars can exhibit different adaptive responses to the same environmental conditions. Indeed, the overall photosynthetic capacity under stress might differ between species or ecotypes and may interact with the severity of the environmental stress. Winter annuals exposed to low temperature exhibited upregulation of photosynthesis, increased carbohydrates, and changes in morphological and anatomical features (Cohu et al., 2014; Muller et al., 2014; Stewart et al., 2016). This study investigated the variation in photosynthetic responses of different *Stevia* cultivars to low temperature, which could be a valuable resource to illustrate the performance and photoinhibition of different cultivars. We exposed nine *Stevia* cultivars to cold stress *in vitro* and measured F_v_/F_m_, PI_ABS_, C_i_, P_N_, WUE, and Chl *a*, Chl *b*, and carotenoid contents. We also measured WSC and glucose accumulation to characterize any correlation between carbohydrate content and photosynthesis. Changes in the morphology and anatomy of stomata and stems were also studied in cold-stressed *Stevia* cultivars.

## Materials and Methods

### Plant Culture and Cold Stress

Nine cultivars of *S. rebaudiana* were obtained from the EUSTAS Stevia Gene Bank and labeled as accessions 1–9 (Laboratory of Tropical Crop Improvement, Leuven, Belgium; Van den Houwe et al., 2011). Three seedlings per cultivar were cultured *in vitro* on Murashige and Skoog (1962) medium in a glass jar. At least 20 glass jars containing three seedlings were prepared for each *Stevia* cultivar. The plants were incubated in a growth chamber set at 25 ± 1°C with a photoperiod of 16 h under white fluorescent lamps (150 μmol photon m^-2^ s^-1^) for 2 months. After 2 months, eight glass jars of each cultivar containing three seedlings of similar height, stem number, and leaf size were selected for the cold stress and control treatments. Four of these jars of each cultivar were maintained in a growth chamber set at 25 ± 1°C with a photoperiod of 16 h under white fluorescent lamps (150 μmol photon m^-2^ s^-1^) while the other four jars were exposed to cold stress for 1 month in a growth chamber set at 5 ± 1°C with the same photoperiod regimes. At the end of the cold stress, all plants were moved to a growth chamber set at 25 ± 1°C for 4 h before undertaking further analysis (photosynthetic efficiency, photosynthetic carbon dioxide uptake, pigment and carbohydrate sampling, and anatomical studies) for a more reliable comparison among treatments and cultivars, with all samples having the same leaf temperature.

### Gas Exchange and Fluorescence

For all treatments, measurements were taken in triplicate on the six youngest fully expanded leaves per plant of four plants from four jars. The measurements were done on these leaves because they had emerged during the treatment. All plants were exposed to light in the growth chamber for about 2 h, before being placed in the dark for at least 30 min prior to obtaining values for F_v_/F_m_ and PI_ABS_ using a portable chlorophyll fluorometer (Pocket PEA, Hansatech, England). The following equations were used to calculate F_v_/F_m_ and PI_ABS_ parameters (Živčák et al., 2008):

$$\begin{array}{c} {\text{F}}_{\text{V}}/{\text{F}}_{\text{M}}=({\text{F}}_{\text{M}}-{\text{F}}_{\text{o}})/{\text{F}}_{\text{M}}\\ {\text{PI}}_{\text{ABS}}=\frac{1-({\text{F}}_{\text{o}}/{\text{F}}_{\text{M}})}{{\text{M}}_{\text{o}}/{\text{V}}_{\text{J}}}\times \frac{{\text{F}}_{\text{M}}-{\text{F}}_{\text{o}}}{{\text{F}}_{\text{o}}}\times \frac{1-{\text{V}}_{\text{J}}}{{\text{V}}_{\text{J}}} \end{array}$$

where F_o_ is fluorescence intensity, F_M_ is maximum fluorescence intensity, V_J_ is relative F_v_, and M_o_ is the initial slope of fluorescence kinetics. Intercellular CO_2_ concentration (C_i_), P_N_, and WUE were measured using a portable plant photosynthesis meter (KR8700 system; Korea Tech Inc., South Korea). All samples had the same leaf temperature. The six youngest fully expanded leaves per plant were then harvested, as one replicate, for the measurements outlined in the following three sections.

### Photosynthetic Pigments

The Chl *a*, Chl *b*, and carotenoid contents were determined using an extraction in 80% (v/v) acetone as per the method of Lichtenthaler and Wellburn (1983).

### Water-Soluble Carbohydrates

Water-soluble carbohydrates (WSC) were determined according to the phenol-sulfuric-acid method (Dubois et al., 1956).

### Glucose

Some of the extract obtained from the phenol-sulfuric-acid method above was used to measure glucose content with a glucose assay kit (Sigma).

### Anatomical Studies

The anatomical characteristics of *Stevia* stems and leaves were evaluated after 1 month of cold stress. Four stem samples from four plants in different jars of each cultivar were fixed in formalin–acetic acid–alcohol (FAA 1:1:18 v/v). Cross-sections were manually cut, double-stained with carmine and methyl green (Amini Rad and Sonboli, 2008), and then viewed and photographed using a light microscope (Olympus BX51) with an automatic camera at 100 × and 400 × magnifications. Anatomical features—stem diameter (mm), epidermal width (μm), dermal width (μm), xylematic vessel width (μm), number of sclerenchyma per colony, sclerenchyma colony width (μm), and phloem tissue width (μm)—were measured using Image Tools Version 3.0 software. The fresh weight and area of the six youngest fully extended leaves per plant were measured, and the fresh leaf mass per area (mg fresh weight cm^-2^), leaf area of six leaves (cm^2^), and fresh mass of six leaves (mg) are reported. For leaf epidermis properties, a colorless nail coating was brushed on the adaxial leaf side of the six youngest fully expanded leaves per plant of each cultivar. The sellotape, with its varnish imprint, was carefully removed from the leaf to obtain epidermal impressions for analysis (Vezza et al., 2018). The number of stomata was measured using an Olympus BX51 microscope at 200× magnification. The length and width of guard cells were measured at 400× magnification with Image Tools Version 3.0 software. Stomatal density (SD) and epidermal cell density (ECD) were measured per unit area and used to calculate the stomatal index (SI):

SI=SD/(SD+ECD)×100

### Statistical Analyses

The experiment (e.g., plant culture and treatment) was repeated three times, with a separate analysis conducted for each experiment. Each experiment included the set of four jars with three plants for each treatment and each cultivar. Statistical analysis was performed with SPSS software (version 23). For the parameters of F_v_/F_m_, PI_ABS_, C_i_, P_N_, and WUE, the average of the collected data for six leaves per plant was considered as one replicate. The means for F_v_/F_m_, PI_ABS_, C_i_, P_N_, and WUE, photosynthetic pigments, WSC and glucose are the average of 12 plants from 12 jars across three experiments. The means of anatomical features are the average of four values from four plants in four jars for each treatment. For leaf biomass per area, the average of six leaves per plant was considered as one replicate, and the means are the average of four individual plants from four jars. The sum of fresh mass and area of six leaves per plant were considered as one replicate and the means are the average of four individual plants from four jars. Significant differences between parameters were determined by two-way ANOVA test. The Duncan’s test (*p* ≤ 0.05) was used to compare means.

## Results

**FIGURE 1:**
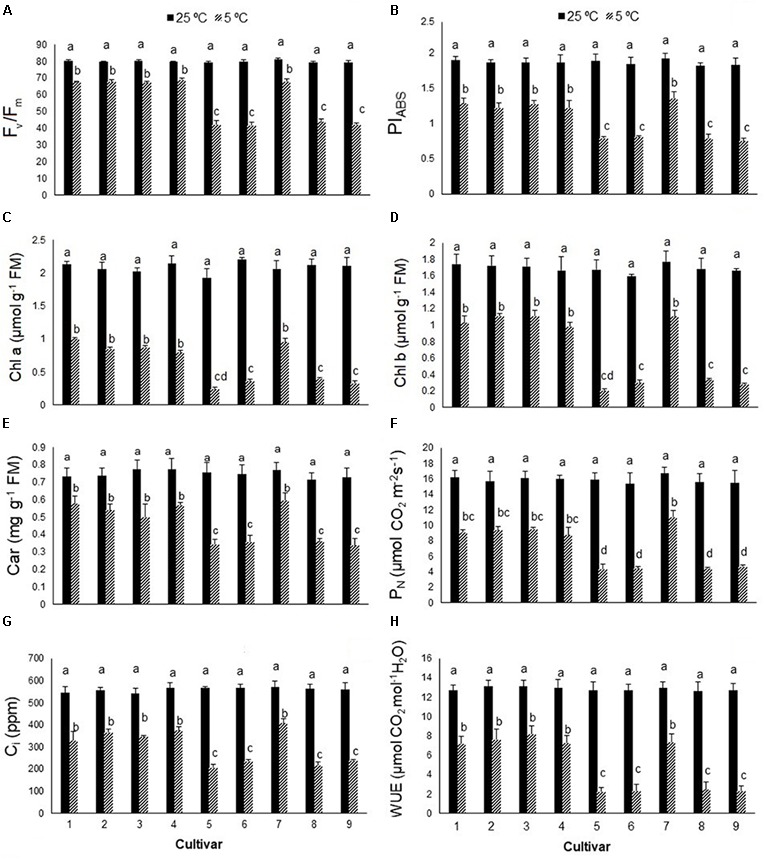
**(A)** The maximum quantum yield of photosystem II (F_v_/F_m_), **(B)** performance index (PI_ABS_), **(C)** chlorophyll *a* (Chl *a*), **(D)** chlorophyll *b* (Chl *b*), **(E)** carotenoids (car), **(F)** net photosynthesis (P_N_), **(G)** intercellular CO_2_ (C_i_), and **(H)** water use efficiency (WUE) in nine cultivars of *S. rebaudiana* exposed to cold stress (5°C) relative to the controls (25°C). The experiment comprised a set of four jars in triplicate for each treatment. Two-way ANOVA was used to determine statistical differences. The means are the average of 12 plants. Columns with the same lower-case letters do not differ significantly at *p* < 0.05; error bars represent standard deviation.

The F_v_/F_m_ ratio of *S. rebaudiana* declined significantly in all cultivars from about 0.80 in control plants to 0.41–0.69 in cold-stressed plants (**Figure 1A**), more so in cultivars 5, 6, 8, and 9 (44–47%) than cultivars 1, 2, 3, 4, and 7 (12–14%). Cold stress significantly reduced the PI_ABS_ value by 50–73% (**Figure 1B**). Photosynthetic pigments (Chl *a*, Chl *b*, carotenoids) also declined with cold stress, more so in cultivars 5, 6, 8, and 9 than cultivars 1, 2, 3, 4, and 7 (**Figures 1C–E**). Cold stress significantly reduced P_N_ by 81–84% in cultivars 5, 6, 8, and 9 and by 46–57% in cultivars 1, 2, 3, 4, and 7, relative to the controls (**Figure 1F**). Cold stress significantly reduced the intracellular CO_2_ (C_i_) concentration (**Figure 1G**) and WUE (**Figure 1H**), with similar reductions in P_N_, PI_ABS_, photosynthetic pigments, and F_v_/F_m_ value in each cultivar.

**FIGURE 2:**
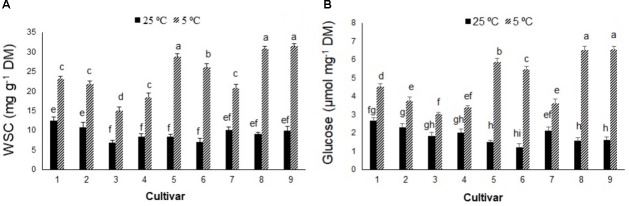
**(A)** Water-soluble carbohydrates (WSC) and **(B)** glucose contents in nine cultivars of *S. rebaudiana* exposed to cold stress (5°C) relative to the controls (25°C). The experiment comprised a set of four jars in triplicate for each treatment. Two-way ANOVA was used to determine statistical differences. The means are the average of 12 values. Columns with the same lower-case letters do not differ significantly at *p* < 0.05; error bars represent standard deviation.

**FIGURE 3:**
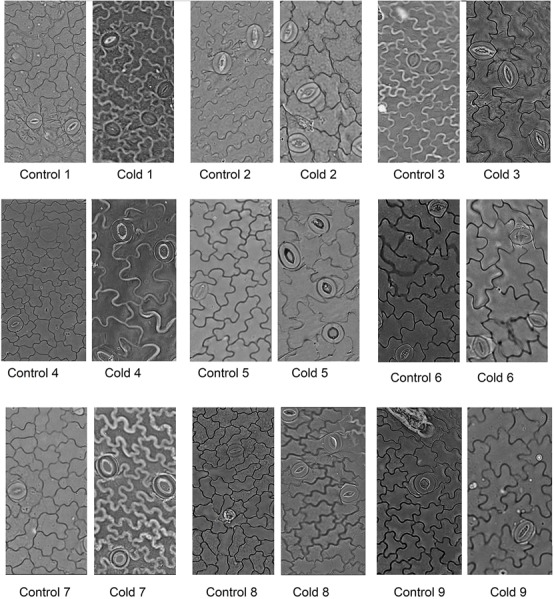
Microphotographs of stomata on the adaxial leaf epidermis of nine cultivars of *S. rebaudiana* in control (25°C) and cold-stressed (5°C) plants.

**FIGURE 4:**
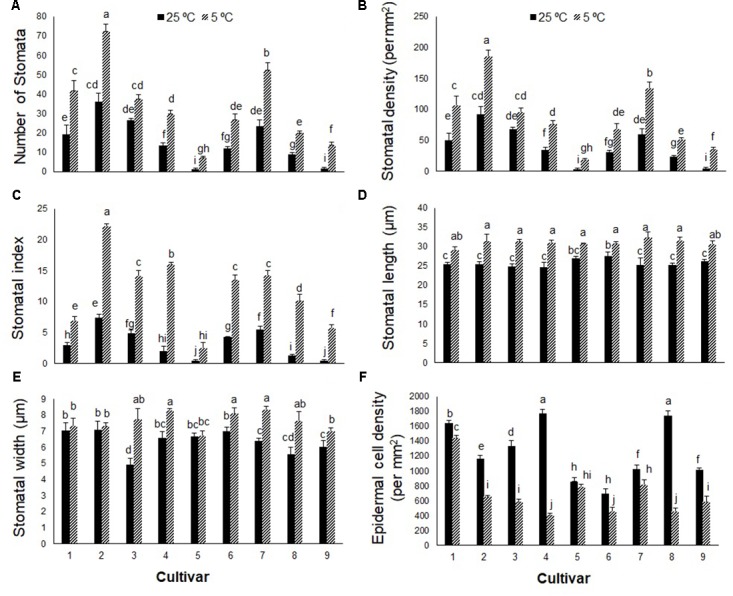
**(A)** Stomatal number, **(B)** stomatal density, **(C)** stomatal index, **(D)** stomatal length, **(E)** stomatal width, and **(F)** epidermal cell index in nine cultivars of *S. rebaudiana* exposed to cold stress (5°C) relative to the controls (25°C). The experiment comprised a set of four jars for each treatment. Two-way ANOVA was used to determine statistical differences. The means are the average of four values. Columns with the same lower-case letters do not differ significantly at *p* < 0.05; error bars represent standard deviation.

**FIGURE 5:**
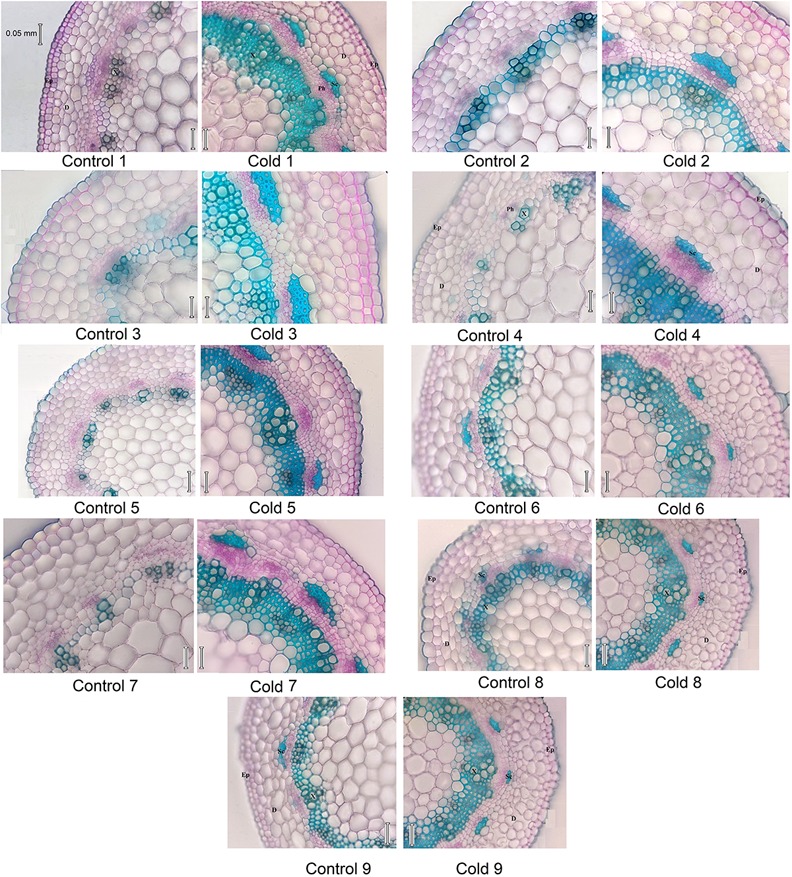
Micrograph of stem cross section in nine cultivars of *S. rebaudiana* in control (25°C) and cold-stressed (5°C) plants. D, Derma; Ep, epiderma; Sc, sclerenchyma; X, xylem vessel.

In contrast to photosynthetic traits, the amounts of WSC and glucose increased significantly in all cultivars in response to cold stress (**Figures 2A,B**), more so in cultivars 5, 6, 8, and 9 (by ∼300–400%) than cultivars 1, 2, 3, 4, and 7 (by ∼100–200%). Cold stress caused morphological and anatomical changes in the stomata of leaves and stems of *Stevia* plants (**Figures 3**, **5**); cultivars 2 and 5 had the highest and lowest stomatal number (72 vs. 7), stomatal density (186 vs. 19), and stomatal index (22 vs. 3), respectively (**Figures 4A–C**). Cold stress significantly increased stomatal length in all cultivars, relative to the controls, while stomatal width increased in cultivars 3, 4, 6, 7, 8, and 9 (**Figures 4D,E**). Cold stress increased epidermal cell size, thus reducing ECD, with the highest and lowest reductions in cultivars 4 and 5, being ∼77 and 8% less than that in their control plants, respectively (**Figure 4F**).

**FIGURE 6:**
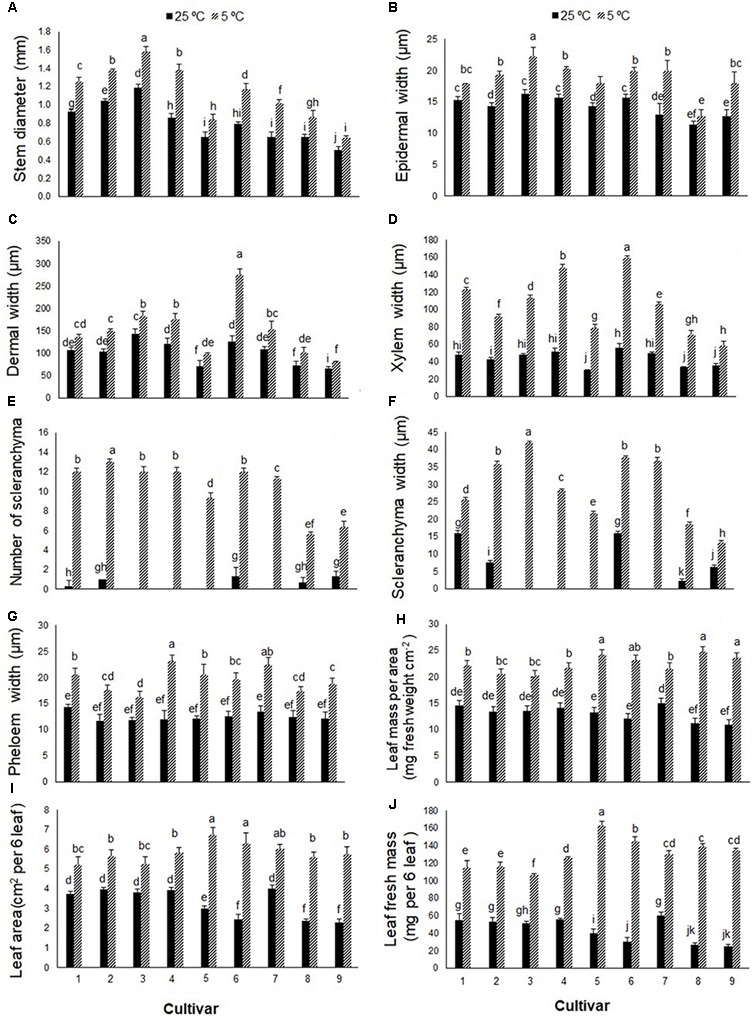
**(A)** Stem diameter, **(B)** epidermal width, **(C)** dermal width, **(D)** xylematic vessel width, **(E)** number of sclerenchyma per colony, **(F)** width of sclerenchyma colony, **(G)** phloem tissue width, **(H)** leaf mass per area, **(I)** leaf area of six leaves, and **(J)** leaf mass of six leaves in nine cultivars of *S. rebaudiana* exposed to cold stress (5°C) relative to the controls (25°C). The experiment comprised a set of four jars for each treatment. Two-way ANOVA was used to determine statistical differences. The means are the average of four values. Columns with the same lower-case letters do not differ significantly at *p* < 0.05; error bars represent standard deviation.

Stem cross-sections differed between cold-stressed and control plants in all *Stevia* cultivars (**Figure 5**). Cold stress significantly increased stem diameter and thickness of epidermal, dermal, and xylematic vessel and phloem areas in all cultivars (**Figures 6A–D,G**). The control plants of cultivars 1, 2, 6, 8, and 9 had few sclerenchyma colonies; cold stress promoted sclerenchyma colony production in all cultivars (**Figure 6E**) and increased the thickness of sclerenchyma (**Figure 6F**). Leaf fresh mass increased significantly in all cultivars in response to cold stress, by about 34, 35, 33, 35, 45, 48, 30, 55, and 54% in cultivars 1 through 9, respectively (**Figure 6H**). The leaf area of six fully expanded leaves increased significantly in all cultivars under cold stress, by about 28, 30, 27, 32, 55, 61, 33, 58, and 60% in cultivars 1 through 9, respectively (**Figure 6I**). Cold stress significantly increased the fresh mass of six leaves in all cultivars with the highest increase in cultivars 5, 6, 8, and 9, by about 76, 80, 81, and 81%, respectively (**Figure 6J**).

## Discussion

In recent years, much attention has been given to changes in photosynthetic traits in response to stress. The F_v_/F_m_ value is a reliable indicator of plant adaptation to stress (Sharma et al., 2012). Chlorophyll fluorescence emission was used by Mishra et al. (2011) to classify cold tolerance in nine *Arabidopsis thaliana* accessions. In this study, an initial screening measured the F_v_/F_m_ value and PI_ABS_ in nine cultivars of *S. rebaudiana* from the EUSTAS Stevia Gene Bank (accessions 1–9) exposed to cold stress. The F_v_/F_m_ values declined in all nine cultivars in response to cold stress, and the magnitude of the reduction was genotype-dependent, being greater in cultivars 5, 6, 8, and 9 than cultivars 1, 2, 3, 4, and 7. Soufi et al. (2016) also reported significant reductions in the F_v_/F_m_ value in *Stevia* plants under cold stress, which may be a protective mechanism of dissipating excitation energy in the PSII antennae as heat (Krause and Laasch, 1987; Adams et al., 2013, 2014) or damage to the Dl protein of PSII reaction centers responsible for photoinhibition (Groom and Baker, 1992; Adams et al., 2013). The PI_ABS_ value provides quantitative information on the current state of plant performance under unfavorable conditions (Strasser et al., 2004). In the current study, the PI_ABS_ value declined significantly in the nine *Stevia* cultivars in response to low temperature, more so in cultivars 5, 6, 8, and 9 than cultivars 1, 2, 3, 4, and 7. Fan et al. (2015) reported lower PI_ABS_ values in cold-stressed Bermudagrass than the control. Adams et al. (2013) revealed that a reduction in photosynthesis might reflect photoinhibition (indicated by a reduction in F_v_/F_m_ and/or reduction in CO_2_ uptake or O_2_ evolution) to prevent photodamage in plants exposed to unfavorable conditions such as high light or low temperature. The decline in PI_ABS_ and F_v_/F_m_ values in stressed *Stevia* plants suggests that the cold stress induced photoinhibition in all cultivars for plant survival and to prevent photodamage or damage to photosynthetic apparatus. Further measurements of photosynthetic pigments, photosynthesis, and carbohydrates provided more evidence for the downregulation of photosynthesis in response to cold stress.

We measured Chl *a*, Chl *b*, and carotenoid contents to characterize the correlation between the quantum efficiency of the photosystems and photosynthetic pigments in the reaction centers; all of which declined significantly under cold stress, more so in cultivars 5, 6, 8, and 9, and is in accordance with reports on rice (Han et al., 2017). In response to cold stress, Chl *a* and *b* contents declined the most in cultivars 9 and 5, by ∼84 and ∼88%, respectively, relative to their controls. Carotenoid content declined the most in cultivar 5, by ∼54% relative to its control. The smaller reduction in carotenoid content than Chl *a* or *b* content in response to cold stress may reflect the role of xanthophyll cycle carotenoids in releasing thermal energy and protecting PSII reaction centers (Demmig-Adams et al., 1996; Adams et al., 2006; Demmig-Adams and Adams, 2006). Tewari and Tripathy (1998) reported that a reduction in Chl biosynthesis in plants exposed to low-temperature stress is partially due to the inhibition of 5-aminolevulinic acid biosynthesis. In the current study, the reductions in Chl *a*, Chl *b*, and carotenoid contents in response to cold stress correlated well with the F_v_/F_m_ and PI_ABS_ parameters. Several studies have demonstrated that low-temperature stress inhibits photosynthesis, as indicated by reductions in photosynthetic rate and pigment contents (Boese and Huner, 1990; Strand et al., 1997). A reduction in photosynthetic pigments reduces the level of light absorption and can be followed by a reduction in the level of excess excitation energy, as an alternative to increasing the level of energy dissipation, which lowers PSII efficiency (Demmig-Adams and Adams, 2006). In the nine *Stevia* cultivars, the reduction in light absorption and increase in energy dissipation seem to be acting concomitantly. Adams et al. (2002) reported that mesophytic species tend to upregulate photosynthetic capacity and increase chlorophyll levels in winter, while several evergreens and conifers tend to upregulate photoprotection and retain chlorophyll, which relies more on higher levels of energy dissipation.

A widely used method for studying plant photosynthesis is P_N_ analysis. Winter annual species exhibited higher photosynthetic rates than summer annual species under cool temperatures (Adams et al., 2013; Demmig-Adams and Adams, 2018). The downregulation of photosynthesis and reduction in P_N_ in the nine *Stevia* cultivars in response to low-temperature conditions is related to the observed reduction in chlorophyll fluorescence. Cold stress significantly reduced P_N_, more so in cultivars 5, 6, 8, and 9 than cultivars 1, 2, 3, 4, and 7, which could be related to changes in fluorescence. Photoinhibition can be characterized as a decrease in photosynthetic CO_2_ uptake (Adams et al., 2013). In the present study, the CO_2_ concentration declined significantly in response to cold stress, which was more pronounced in cultivars 5, 6, 8, and 9 than cultivars 1, 2, 3, 4, 7.

The D1 protein of the PSII reaction center is involved in water oxidation to provide electrons for PSI. Photoinactivation or photodamage of the D1 protein during photoinhibition is a mechanism responsible for photoprotection (Adams et al., 2006, 2013). Damage to the D1 protein and oxygen-evolving complex is followed by a reduction in water oxidation (Adams et al., 2013). A decline in water status in response to cold stress may play an indirect role by reducing the capacity for gas exchange. In our study, the WUE of *Stevia* leaves declined significantly at 5°C, more so in cultivars 5, 6, 8, and 9 than cultivars 1, 2, 3, 4, and 7. The reduction in leaf water status was linked to lower CO_2_ concentrations and a reduction in photosynthesis. Often, increases in intercellular CO_2_ concentration are beneficial for photosynthesis, while decreases suggest inhibition of the photosynthetic apparatus. Zhu et al. (2018) reported that CO_2_ elevation enhanced the efficiency of photosynthetic electron transport in cold-stressed wheat plants. Thus, in our study, the reduction in gas exchange capacity of the *Stevia* plants exposed to cold stress was likely followed by a reduction in P_N_, which supports the findings of Soufi et al. (2016) that cold stress had a negative effect on photosynthesis in *Stevia* plants.

The light energy absorbed by Chl molecules can be used in photosynthesis (sugar biosynthesis), dissipated as heat, or re-emitted as Chl fluorescence (Maxwell and Johnson, 2000). Sugar synthesis occurs in a plant’s mature source leaves, which is then exported to the sinks (e.g., young leaves, fruits) (Adams et al., 2014). Despite the decline in F_v_/F_m_ values and corresponding reductions in P_N_, cold stress increased carbohydrate and glucose accumulation in the nine *Stevia* cultivars, particularly cultivars 5, 6, 8, and 9. Previous reports have shown that low temperatures increase soluble sugars in the leaves of different plants (Strand et al., 1997; Adams et al., 2013, 2014). Modifications to carbohydrate metabolism could be induced by altering the balance between source and sink organs (Adams et al., 2013, 2014; Lemoine et al., 2013). Plants accumulate sugars in source leaves when production exceeds the rate of export (Adams et al., 2013, 2014). Adams et al. (2014) suggested that carbohydrate accumulation in photoinhibited leaves under stress conditions may lead to feedback inhibition, downregulation of photosynthesis, or inhibition of foliar export of carbohydrates. The movement of liquid water out of cells and increased osmotic concentration inside cells prevent intracellular freezing (Ameglio et al., 2001). In wheat, cold stress enhanced leaf vacuolar carbon storage through increased fructans accumulation and the polymerization of sucrose to fructans in the crown tissues (Leonardos et al., 2003). Furthermore, increased amounts of carbohydrate and the subsequent reduction in osmotic potential have been observed in cold-stressed (5°C) wheat plants (Equiza et al., 2001). A linear correlation between carbohydrate accumulation and SVglys levels has been reported in the leaves of *S. rebaudiana* (Ceunen and Geuns, 2013), so the higher glucose content in cold-stressed *Stevia* cultivars may be correlated with higher SVglys biosynthesis.

In addition to the increased carbohydrate contents in the nine *Stevia* cultivars, the amount of phloem tissue in the stem increased significantly in response to cold stress, which may be needed to export the greater volume of carbohydrates produced in leaves to the rest of the plant. Similarly, Cohu et al. (2014) reported a significant increase in sugar content and phloem tissue in the leaf veins of *Spinacia oleracea* and *A. thaliana* in response to cool temperatures. Furthermore, the higher leaf mass per unit area in response to cold stress may reflect increased cell wall, as reported by Domon et al. (2013) in *Miscanthus* ecotypes. *Stevia* leaves are used as a natural sweetener (Geuns, 2003), so higher leaf mass and carbohydrate contents in cold-stressed *Stevia* cultivars can reflect higher yields at low temperature. *S. rebaudiana* originated in Paraguay with an optimum growth temperature of 24°C (Ahmed et al., 2011), yet all nine cultivars of *Stevia* in this study produced more leaf mass and area under cold stress. Cold stress increased fresh leaf mass, carbohydrate contents, phloem tissue, and stem diameters in the nine *Stevia* cultivars regardless of the observed reductions in photosynthetic capacity, which possibly reflects plant adjustment to cold stress.

There are reports on the effect of cold stress on stomatal apparatus and epidermal cells (Boese and Huner, 1990; Ameglio et al., 2001). In the present study, cold stress significantly affected leaf and stem anatomy in the *Stevia* cultivars, increasing the stomatal number, size, density, and index, and decreasing ECD. In spinach, stomatal number and ECD did not change when exposed to low temperature (5°C) stress (Boese and Huner, 1990). In *Stevia*, the increase in stomatal index and density could increase water loss and reduce leaf water potential, which may explain the observed reductions in WUE, C_i_, and P_N_ in the nine tested cultivars. Some plants exhibit low leaf water potentials in response to chilling stress, which may limit photosynthetic activity (Pardossi et al., 1992; Wilkinson et al., 2001). Cold stress increased the width of derma, epiderma, xylem, phloem, and sclerenchyma, which was followed by an increase in stem diameter in all *Stevia* cultivars. Equiza et al. (2001) suggested that increased epidermal thickness in response to cold stress is an adaptive reaction to increase freezing tolerance. Sclerenchyma is one of the main supporting tissues of growing plant organs (Leroux, 2012); its increase in response to cold stress may have increased stem hardness in the *Stevia* cultivars. The increase in xylem vessels in response to cold stress may increase water transport from roots to leaves to prevent reductions in water potential. The diameter and frequency of xylem vessels are critical determinants of water conductance (Lovisolo and Schubert, 1998).

## Conclusion

Cold stress significantly reduced the measured photosynthetic traits – F_v_/F_m_, PI_ABS_, P_N_, WUE, C_i_, and Chl *a*, Chl *b*, and carotenoid contents – in *Stevia* cultivars, but the impact was more evident in cultivars 5, 6, 8, and 9 than cultivars 1, 2, 3, 4, and 7. The degree of reduction in the measured traits was related to genotype. Regardless of the observed reduction in photosynthetic capacity, the carbohydrate content and leaf mass and area increased, more so in cultivars 5, 6, 8, and 9. These results suggest downregulation of photosynthetic activity by photoinhibition to increase photoprotection of the photosynthetic apparatus. Even though all Stevia cultivars exhibited photoinhibition under cold stress, the higher carbohydrate accumulation and leaf mass produced at 5°C, compared to 25°C, suggests no critical damage of photosystems being responsible for the reduction in chlorophyll fluorescence and photosynthesis. Measuring SVglys content, a critical characteristic of *S. rebaudiana* cultivars, to identify their absolute yield in regions with cold winters. Furthermore, the upregulation of anatomical features in *Stevia* plants may contribute to the success of all cultivars under cold stress. In future, more detailed measurements – including photosynthetic parameters (non-photochemical quenching, zeaxanthin, D1 protein, transpiration), reactive oxygen species, antioxidant system, quantitative analyses of responsible genes in SVglys biosynthesis, carbohydrate metabolism, and sugar exudation – could help to determine how plants contribute to cold stress. Furthermore, a complementary study on the lethal temperature 50 (LT50) of plants and their performance after the transition of cold-stressed *Stevia* cultivars to warmer temperatures would help to identify more cold-adapted cultivars.

## Author Contributions

SH, FN, JG, ID, and KS designed the research and wrote the manuscript. SH did the experiments and analyzed the results. SH and FN did the anatomical experiments. JG provided the plants.

## Conflict of Interest Statement

The authors declare that the research was conducted in the absence of any commercial or financial relationships that could be construed as a potential conflict of interest.

## Abbreviations

car: carotenoids
Chl: chlorophyll
C_i_: intercellular CO_2_
F_m_: maximum fluorescence
F_v_: variable fluorescence
F_v_/F_m_: maximum photochemical efficiency of PSII
PI_ABS_: performance index
P_N_: net photosynthesis
SVglys: steviol glycosides
WSC: water-soluble carbohydrates
WUE: water use efficiency
